# Effect of Ratio in Ammonium Nitrate on the Structural, Microstructural, Magnetic, and AC Conductivity Properties of BaFe_12_O_19_

**DOI:** 10.3390/ma11112190

**Published:** 2018-11-06

**Authors:** Raba’ah Syahidah Azis, Nor Nadhirah Che Muda, Jumiah Hassan, Abdul Halim Shaari, Idza Riati Ibrahim, Muhammad Syazwan Mustaffa, Sakinah Sulaiman, Khamirul Amin Matori, Yap Wing Fen

**Affiliations:** 1 Department of Physics, Faculty of Science, University Putra Malaysia, 43400 UPM Serdang, Selangor, Malaysia; jumiah@upm.edu.my (J.H.); ahalim@upm.edu.my (A.H.S.); mm_syazwan@upm.edu.my (M.S.M.); khamirul@upm.edu.my (K.A.M.); yapwingfen@upm.edu.my (Y.W.F.); 2 Materials Synthesis and Characterization Laboratory, Institute of Advanced Technology (ITMA), University Putra Malaysia, 43400 UPM Serdang, Selangor, Malaysia; idzariati@upm.edu.my (I.R.I.); sakinah6335@yahoo.com (S.S.)

**Keywords:** Barium ferrites (BaFe_12_O_19_; BF), steel wastes, ammonium nitrate salt melts method (ANSM), microstructures, magnetic properties, AC conductivity

## Abstract

This paper investigates the effect of the ratio of ammonium nitrate (AN) on the structural, microstructural, magnetic, and alternating current (AC) conductivity properties of barium hexaferrite (BaFe_12_O_19_). The BaFe_12_O_19_ were prepared by using the salt melt method. The samples were synthesized using different powder-to-salt weight ratio variations (1:3, 1:4, 1:5, 1:6 and 1:7) of BaCO_3_ + Fe_2_O_3_ and ammonium nitrate salt. The NH_4_NO_3_ was melted on a hot plate at 170 °C. A mixture of BaCO_3_ and Fe_2_O_3_ were added into the NH_4_NO_3_ melt solution and stirred for several hours using a magnetic stirrer under a controlled temperature of 170 °C. The heating temperature was then increased up to 260 °C for 24 hr to produce an ash powder. The x-ray diffraction (XRD) results show the intense peak of BaFe_12_O_19_ for all the samples and the presence of a small amount of the impurity Fe_2_O_3_ in the samples, at a ratio of 1:5 and 1:6. From the Fourier transform infra-red (FTIR) spectra, the band appears at 542.71 ${\mathrm{cm}}^{-1}$ and 432.48 ${\mathrm{cm}}^{-1}$, which corresponding to metal–oxygen bending and the vibration of the octahedral sites of BaFe_12_O_19_. The field emission scanning electron microscope (FESEM) images show that the grains of the samples appear to stick each other and agglomerate at different masses throughout the image with the grain size 5.26, 5.88, 6.14, 6.22, and 6.18 µm for the ratios 1:3, 1:4, 1:5, 1:6, and 1:7 respectively. From the vibrating sample magnetometer (VSM) analysis, the magnetic properties of the sample ratio at 1:3 show the highest value of coercivity *H*_c_ of 1317 Oe, a saturation magnetization *M*_s_ of 91 emu/g, and a remnant *M*_r_ of 44 emu/g, respectively. As the temperature rises, the AC conductivity is increases with an increase in frequency.

## 1. Introduction

Barium hexaferrites (BaFe_12_O_19_) have a hexagonal magnetoplumbite structure [1] and they are considered to be unique materials. They are hexagonal, with space group *P6_3_*/*mmc* (No. 194) with lattice dimensions a = b ≈ 5.90 Å, c ≈ 23.30 Å containing 38 oxygen ions, 24 iron ions, and two barium ions [2,3]. This is due to its interesting properties, such as high chemical stability [4], large magnetocrystalline anisotropy [1], large saturation magnetization [5], high coercivity [1], and high resistivity [6]. In contrast to other complex iron oxides, i.e., orthoferrites and spinel-ferrites, the real part of the dielectric constant for diamagnetically substituted hexaferrites decreases more slowly at low frequencies, and almost monotonically with diamagnetic substitution. The real and imaginary parts of the permeability have a peak near 50 GHz, which is determined by the level of diamagnetic substitution [7]. BaFe_12_O_19_ ferrites are used in many aspects of technology, such as high frequency microwaves [8,9,10], magnetic recording media [11], permanent magnets, magnetic sensor applications [12], sensors [13,14,15], etc. Apart from that, BaFe_12_O_19_ ferrites are also used in water purifiers to separate the paramagnetic minerals and to eliminate some of metallic impurities in water [16]. Steel wastes such as steel scraps or mill scales are waste materials that form on the surface of the steel during the steel making process [17]. Mill scales are attractive industrial wastes due to their high iron content of about 72%, low levels of impurities, and stable chemical composition [18,19,20]. They consist of wuestite (FeO), hematite (α-Fe_2_O_3_), and magnetite (Fe_3_O_4_) [17]. High purities of α-Fe_2_O_3_ can be produced from the mill scale separation process [20]. Hematite, which is also known as iron (III) oxide (α-Fe_2_O_3_), has a hexagonal close packing of O_2_, with 2/3 of the interstices being filled with Fe^3+^, and each cation being surrounded by six O_2_ ions [1]. With the consideration of its high iron composition [18], steel wastes can be used to produce Fe_2_O_3_, which is used as a raw material in ferrite production. Recent studies have also recently discovered the large spontaneous polarization and multiferroic properties at room temperature in barium hexaferrites substituted by diamagnetic cations. Herewith, the magnetoelectric characteristics of M-type hexaferrites fabricated by a modified ceramic technique are more advanced than those for the well-known room temperature BiFeO_3_ orthoferrite, multiferroic [21].

Many techniques were reported for the synthesis of BaFe_12_O_19_. These include sol gel [22], hydrothermal [23], high energy ball milling [24,25], and conventional solid reaction methods [26,27,28] and etc. The conventional solid-state reaction method of preparing BaFe_12_O_19_ by mixing oxides and carbonates have some disadvantages, such as the production of chemical inhomogeneity, and the introduction of impurities during the milling process [25]. The properties of ceramic ferrite materials are known to be strongly influenced by their composition and microstructures that are sensitive to the processing method [29]. Salt melt synthesis is a modification of the powder metallurgical method, in which salt with a low melting point is added to the reactants and heated above the melting point of the salt [30]. This synthesis begins with liquid phase solution or wet-chemical synthesis, and utilizes a solution medium, in which the target materials are generated from a series of chemical and physical transformations. Compared to solid-state reactions for which the rates are usually limited by the slow diffusion of the reactants, the reaction temperature of salt melt synthesis is lower, as it allows for faster mass transfer transport in the liquid phase by the means of convection and diffusion. Solvation is a crucial step for the solvent-based synthetic routes, but molecular solvents hardly solvate many inorganic-like metals and oxides. However, destabilization of metallic, ionic, or covalent bonds by solvent interactions becomes possible at high temperatures in the presence of strong polarizing forces, which can be provided by salt melts—a pool of ionized cations and anions. On the other hand, as many types of salts dissolve in water by nature, salt melt synthesis (SMS) has the advantage of easy isolation of the product. Salt melt systems have been used as a reaction media for the growth of a single crystal [31], high temperature liquid batteries electrolytes [32], high-energy rechargeable metallic lithium batteries [33], and in the electrolysis of refractory metals [33,34,35]. An oxygen-rich precursor is obtained by heating the mixture of ammonium nitrate solution and iron oxide at 260 °C, which is suitable for the formation of complex oxides [36]. The parallel relationship of powder-to-salt ratio, and the magnetic properties of hexagonal ferrite are optimized and closely observed. The ratio of AN salts to oxide during the synthesis of the materials gives effects on the morphology of the samples [37]. According to previous reports on barium ferrite synthesis, none of these studies reported on preparing BaFe_12_O_19_ using the salt melt method. The specific purpose of this research study is to observe the effect of the ratios of ammonium nitrate salt on the structural, microstructural, magnetic, and AC conductivity properties of hexagonal BaFe_12_O_19_, using a new approach of chemical route.

## 2. Materials and Methods

### 2.1. Synthesis of BaFe_12_O_19_ via the Salt Melt Method

The BaFe_12_O_19_ powder was prepared using the ammonium nitrate salt melt method. The barium carbonate, BaCO_3_ (SYSTERM.98%), and ammonium nitrate, NH_4_NO_3_ (AN) (SYSTERM) reagents used were of analytical grade from Systerm without any further purification. The raw material Fe_2_O_3_ were synthesized and reutilized from steel waste products using a magnetic separation technique as previously reported by [38,39]. The calculations of the masses of the sample composition components, and the formation of the weights were performed according to the stoichiometric proportion of the general equation of reaction (Equation (1)):

BaCO_3_ + 6Fe_2_O_3_ → BaFe_12_O_19_ + CO_2_ (1)

The chemicals were mixed with powders to salt molar ratios of 1:3, 1:4, 1:5, 1:6, and 1:7. The ammonium nitrate salts were added into a beaker and heated until melted at 170 °C. The mixed Fe:Ba powders were added into a beaker with melted ammonium nitrate and kept at room temperature. Fe_2_O_3_, BaCO_3_ powder, and ammonium nitrate NH_4_NO_3_ salt (AN) were varied from 1:3 to 1:7. The mixed solution was kept stirred on the hotplate at 260 °C, to form a precipitate on the surface of the glass beaker. The precipitate was dried on the hotplate until a brown-reddish ash was formed. The powders were added with the binder polyvinyl alcohol (PVA) at 1 wt %, and pressed at 5 tons to form pellets. The pellets were finally sintered at 1300 °C for 6 hr, and were then ready for characterization.

### 2.2. Characterizations of BaFe_12_O_19_

The phase identification for the sintered sample was examined with X-ray diffraction, XRD (Philip Expert Pro PW3040) (Grovewood Road, Malvern, UK). The XRD scan ranged from 20 to 80°, with a 0.016° step size at room temperature, operating at 40 kV and 30 mA using CuKα (0.154 nm). The microstructure was observed through a FEI NOVA NanoSEM 230 Field Emission Scanning Electron Microscope, FESEM (Kensington, Sydney, Australia), and the grain size was measured by the mean linear intercept method, involving over 150 grains. The Fourier infrared spectra (200–4000 cm^−1^) was recorded using a Fourier transform infra-red (FTIR) spectrometer (Perkin Elmer model 1650) (Winter Street, Waltham, MA, USA) to determine the infrared spectrum of absorption and emission bands of the sample. The density (*ρ*_exp_) was measured using the Archimedes method, with water as the liquid medium. The theoretical density, *ρ*_xrd_ was calculated using the following equation (Equation (2)):

(2) $$\rho_{\mathrm{xrd}}=\frac{2M}{N_{a}V_{\mathrm{cell}}}$$

where the *ρ*_xrd_ is the theoretical density, the constant 2 represents the number of formula units in a units cell, *M* is the molar mass, *N*_a_ represents Avogadro’s number, and *V*_cell_ is the volume of unit cells which were calculated using the equation (Equation (3)):

(3) $$V_{\mathrm{cell}}=\frac{\sqrt{3}}{2}a^{2}c$$

where *a* and *c* are the lattice constants calculated by indexing the X-ray diffraction (XRD) pattern.

The experimental densities observed were smaller compared to that of the calculated X-ray density, due to the particle coarsening and densification process during the heating process [24]. The relative density and porosity values were calculated using the relation given by Equation (4) [40]:

*ρ*_xrd_ = 8M/N_a_a^3^

*ρ_r_* = [(*ρ*_exp_/*ρ*_xrd_)] × 100% (4)

where *ρ_r_* is the relative density, *ρ*_xrd_ is the X-ray density, M is the molecular weight of a sample, N_a_ is Avogadro’s number, and ‘*a*’ is the lattice constant, which was calculated by indexing the XRD pattern.

The percentage of the porosity of the sintered samples was calculated using the relation (Equation (5)):

(5) $$\mathrm{Porosity}\;(\%\;P)=\left(1-\frac{\rho_{\exp}}{\rho_{theoretical}}\right)\times 100\%$$

where *ρ*_exp_ is the experimental density determined from the Archimedes principle.

The magnetic properties were measured by a vibrating sample magnetometer (VSM) Model 7404 LakeShore (Westerville OH, Paris, France). The measurement was carried out at room temperature. The external field applied was 12 kOe parallel to the sample. The Curie temperature of the samples was measured using the Precision LCR Meter H284A Hewlett Packard (Petaling Jaya, Selangor, Malaysia). The sample was wound with 10 turns of copper wire. Then, the sample was placed in a muffle furnace, and the experiment was conducted, starting from room temperature to 600 °C. The value of inductance, *L*_s_, against temperature, *T*, with an increment of 30 °C, was recorded from the LCR meter at a frequency of 10 kHz. Electrical conductivity properties were measured on the sintered pellets using an Agilent HP4294A High Precision Impedance Analyzer, (Petaling Jaya, Selangor, Malaysia) and an LT furnace. To ensure that the sample made good electrical contact with the electrodes of the sample holder, silver paint was applied on both surfaces of the pellets. The conductivity properties of material were measured in a frequency range from 40 Hz to 1 MHz, and in a narrow temperature range of 30 to 180 °C. Electrical AC conductivity (*σ*_AC_) was quantified by electrical conduction, due to an applied AC field through the samples. The *σ*_AC_ was found to follow the unique conductive properties of a semiconductor at higher temperatures. The *σ*_AC_ can be described via Jonsher’s universal power law [41,42] (Equation (6)):

(6) $$\sigma_{T}(\omega )=\sigma_{dc}(0)+A\omega^{n}$$

where *σ_dc_* is the DC conductivity, which is the frequency-independent conductivity. The AC conductivity *σ*_ac_ obeys the Almond–West universal power law described as Equation (7):

(7) $$\sigma_{\mathrm{ac}}(\omega )=A\omega^{n}$$

where *A* is the AC coefficient, which is a temperature-dependent quantity, and *n* is the frequency exponent of the mobile ions, with the range 0 < *n* < 1. The quantity *σ*_ac_ (*ω*) is the frequency-dependent conductivity used for analyses. The AC conductivity is calculated using the dielectric parameters Equation (8):

(8) $$\sigma_{\mathrm{AC}}=\omega \varepsilon_{o}\varepsilon_{r}{}^{"}$$

where *ω* is the angular velocity, *ε*_o_ is the permittivity of free space, and *ε*_r_^″^ is the dielectric loss factor calculated using Equation (9):

(9) $$\varepsilon_{r}{}^{"}=\frac{Gd}{\varepsilon_{o}\omega A}$$

where *G* is the conductance, *d* is the thickness, and *A* is the cross sectional area of the samples.

## 3. Results and Discussion

### 3.1. Structural Analysis

**Figure 1:**
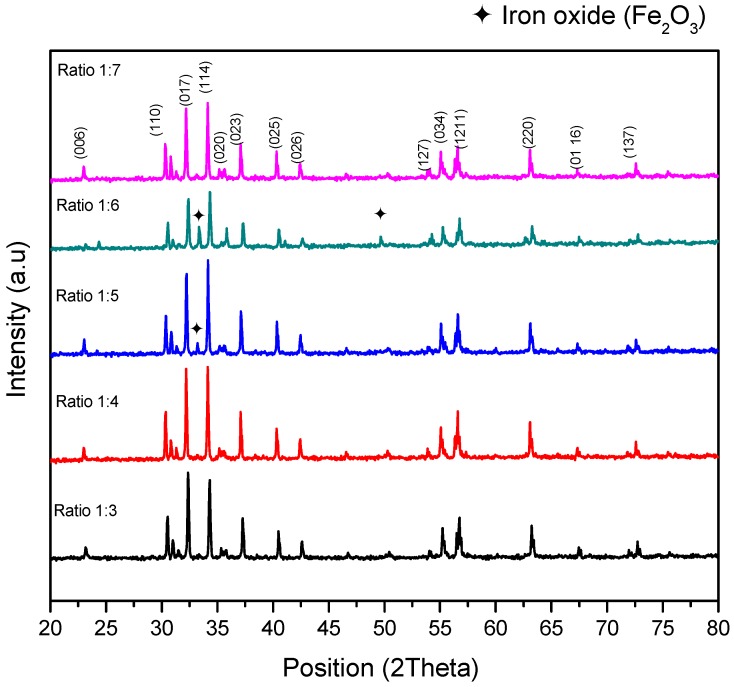
X-ray diffraction pattern for BaFe_12_O_19_ at different ratios of ammonium nitrate.

The XRD spectra of the sintered BaFe_12_O_19_ of various powders to salt ratios are given in Figure 1. The observed peaks corresponded to the formation of the BaFe_12_O_19_ structure for all ratios. Therefore, based on the XRD spectra, BaCO_3_ could be decomposed and BaFe_12_O_19_ could be formed through the salt-melt synthesis method by the diffusion of Ba^2+^, Fe^3+^, and O^2−^ ions with subsequent sintering, without going through calcination or pre-sintering treatments. The peaks were observed and indexed to the ICSD reference code of 98-001-9939. Increasing the amount of AN contents from 1:5 to 1:6 ratios led to an increase in the amount of Fe_2_O_3_ phase. Well-defined sharp peaks indicate the good crystalline quality of the samples [24,43,44].

However, the intermediate phase, Fe_2_O_3_, was observed for powders having ratios of 1:5 and 1:6, and the diffraction planes matched well with JCPDS card no. of 98-004-6404 (Table 1). This was due to incomplete reactions and the homogenization process that occurs in the samples, even when a higher temperature is applied [45]. The crystal structure of BaFe_12_O_19_ could be described by two space groups, the classical *P6_3_*/*mmc* (No.194) and *P6_3_mc* (No.186) space groups. The first and second space groups were used for describing the crystal structure to explain the existence of non-zero spontaneous polarization. This was a polar centrosymmetric and non-centrosymmetric space group [2].

Furthermore, the presence of Fe_2_O_3_ was possibly due to the solubility rates of oxide, which are generally low, while that of carbonates is high [14,15,30,46]. Besides, it was observed from the spectra that the intensity of the peaks for samples having ratios of 1:5 and 1:6 were relatively lower than the other samples. The reduction of the peak’s intensity was prominent in sample having a ratio of 1:6, which in the presence of Fe_2_O_3_ was relatively high. By further increasing the amount of AN content resulted in compositional homogeneity, which only existed in the BaFe_12_O_19_ phase as the major phase in the sample, having a ratio of 1:7, though the degree of crystallinity was not as high as in samples with ratios of 1:3 and 1:4. The BaFe_12_O_19_ peaks corresponded to the diffraction planes (110), (008), (112), (017), (114), (020), (018), (023), (025), (026), (127), (034), (1211), (220), (0116), and (137), respectively, with hexagonal structures [13,40].

The characteristics of the lattice constants *a* and *c* of hexagonal ferrites were calculated according to (Equation 10) [47]:

(10) $$\frac{1}{d^{2}}=\frac{4}{3}\left(\frac{h^{2}+hk+k^{2}}{a^{2}}\right)+\frac{1}{c^{2}}$$

where (*hkl*) are the miller indices, while *d* is the interplanar distance given by the Bragg formula, 2dsinθ=nλ. The volume of the unit cell for all samples was obtained using *a* and *c* parameters according to the relation in (Equation 9). The variations of structural parameters, such as the lattice parameter, unit cell volume, X-ray density, and porosity are shown in Table 2. The obtained lattice parameters *a*, *c*, and the volume of the unit cell (Table 1) were in good agreement, as reported by [1,48], which shows the perfectness of the crystal structure of BaFe_12_O_19_. The X-ray density, *ρ*_xrd_, of this sample, was higher than for single crystals [1], which was due to the larger lattice constant (*a*) obtained from this work.

**Figure 2:**
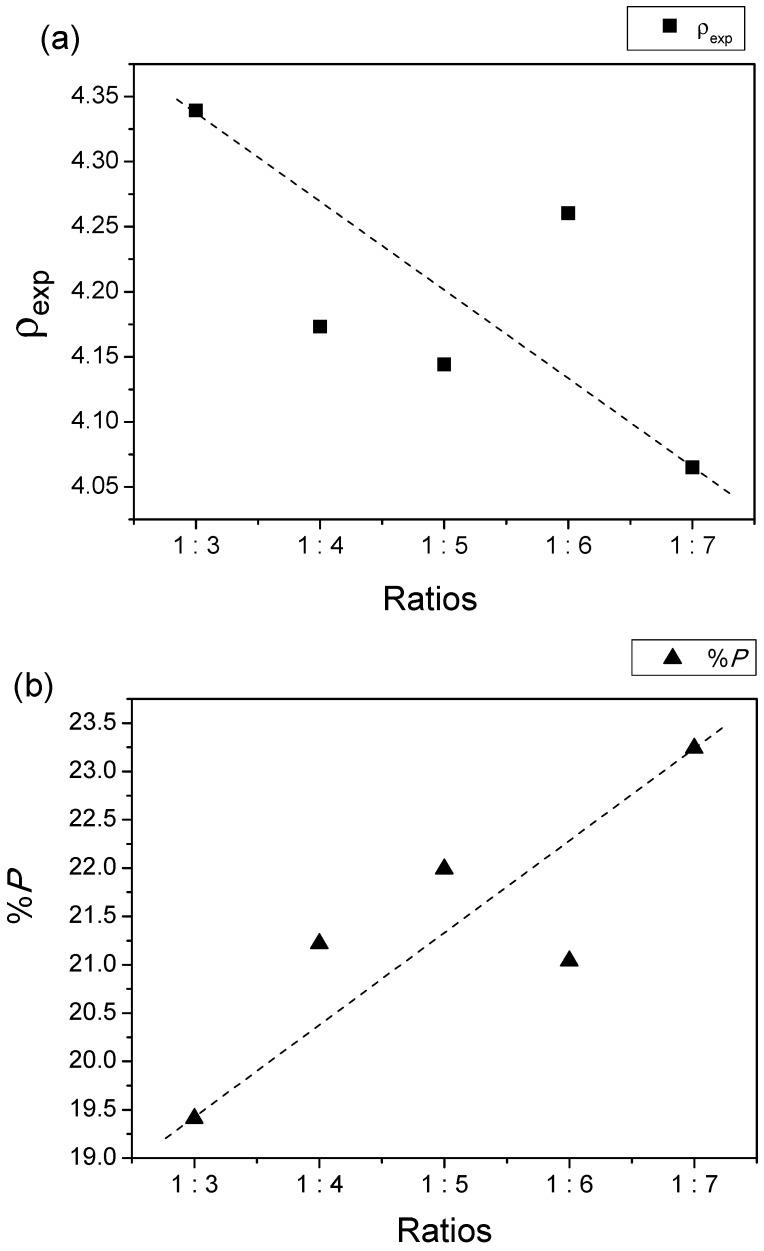
Relationships of (**a**) density, *ρ*_exp_ and (**b**) Percentage of porosity, *%P* of BaFe_12_O_19_.

The values of both lattice parameters (*a* and *c*) change with increase in temperature. This could be attributed to Fe^3+^ in octahedral coordination, which has a larger ionic radius than Fe^3+^ in tetrahedral and pentahedral coordinations, but Fe^2+^ has a larger ionic radius than Fe^3+^ in the same coordination. [49]. In addition, this phenomenon was due to the microstructural defects and interactions between cations [50]. The density values decreased as the AN contents increased (Figure 2a) while the porosity of the samples increased from 19.41% to 23.24% as the AN salts increased (Figure 2b). The nitrate decomposition and gas release during the sintering process led to the formation of the porous volume. The stability of the AN salts significantly affected various parameters, such as the particle granulation, particle size distribution, bulk density, porosity, etc.

A large amount of pores led to a decrease in sample density values [14]. It is important to pay attention to the amount of porosity in the BF system, because a higher amount of porosity would result in more pinning centers to the movement of the domain walls, thus increasing the demagnetizing effects in the sample, and reducing its magnetic properties. The influence on magnetic properties will be discussed in details in the magnetic properties section.

**Figure 3:**
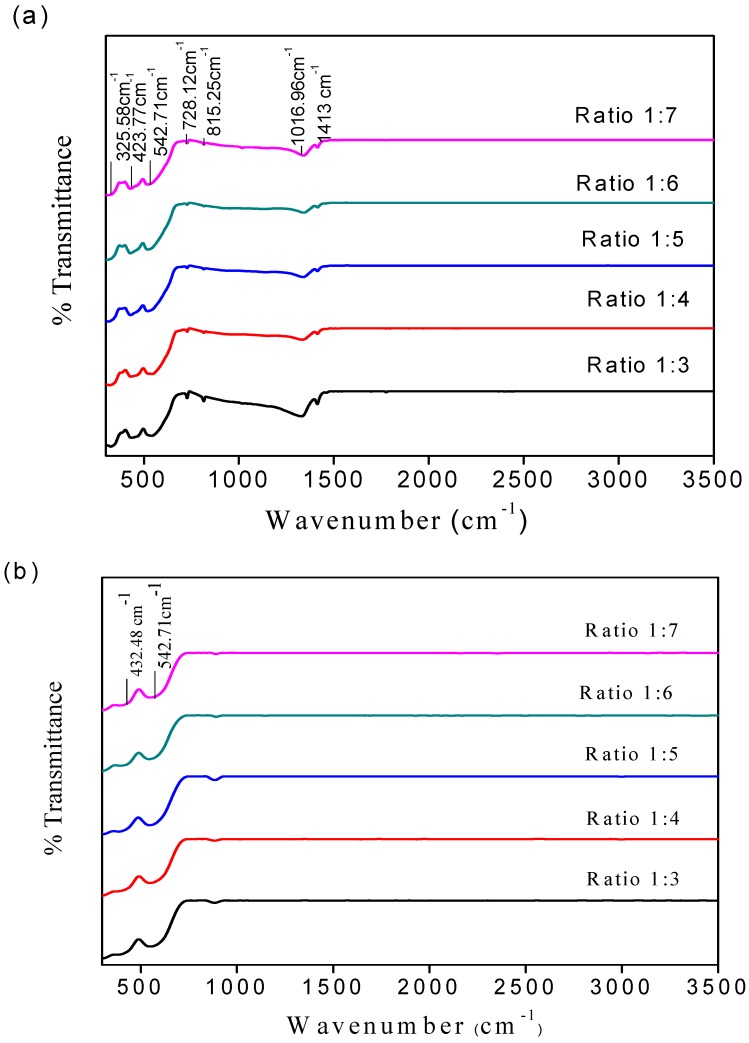
FTIR of BaFe_12_O_19_ (**a**) before sintering and (**b**) after sintering at 1300 °C.

The FTIR spectra of BaFe_12_O_19_ before and after sintering are shown in Figure 3a,b. The FTIR spectra show the characteristic peaks in the required region, which are 1413, 815.25, 542.71 and 432.48 cm^−1^. From Figure 3a, it can be seen that the FTIR characteristics transmittance absorption peak at 1413 cm^−1^ was attributed to C–O stretching, CH_2_ stretching, and C=O [51,52]. The absorption bands at 815.25 cm^−1^ and 728.12 cm^−1^ were assigned to the N–O bending vibration of NO_3_^−^, and to C–N stretching from the ammonium nitrate used in the reaction. [51,52,53].

Figure 3b shows the FTIR characteristic of the absorption band of BaFe_12_O_19_ appearing at 542.71 cm^−1^ and 432.48 cm^−1^ [54,55], which corresponds to metal–oxygen bending and the vibration of octahedral sites of BaFe_12_O_19_ (Table 3).

The vibration wavenumbers are in agreement with previous reports by Mali et al. [53] and Mandizadeh et al. [54]. This shows that there were no other elements, such as ammonia, that were used in the experiment. All of the sample series exhibited typical M-type hexaferrite spectra.

### 3.2. Microstructural Analysis

**Figure 4:**
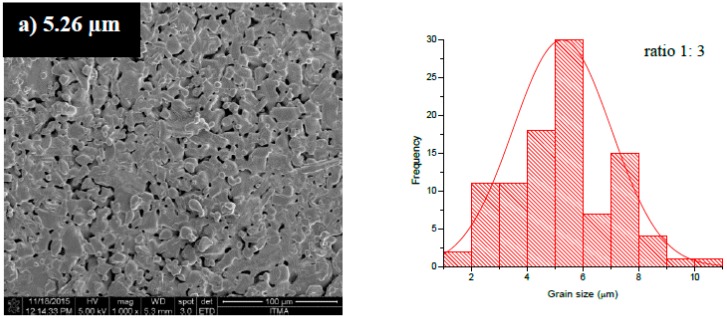

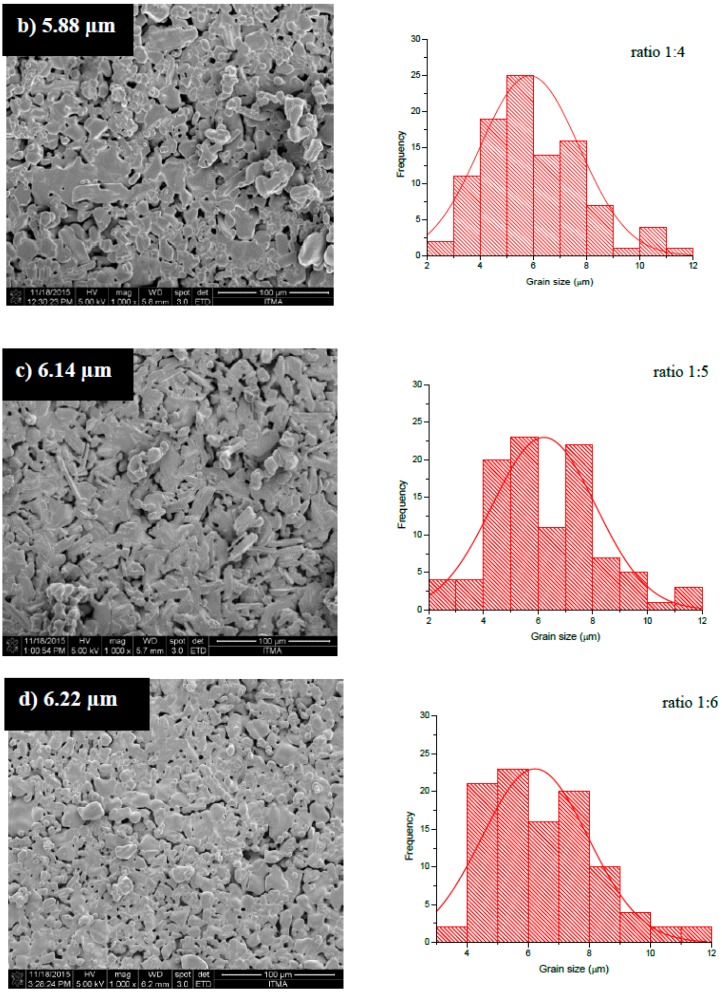

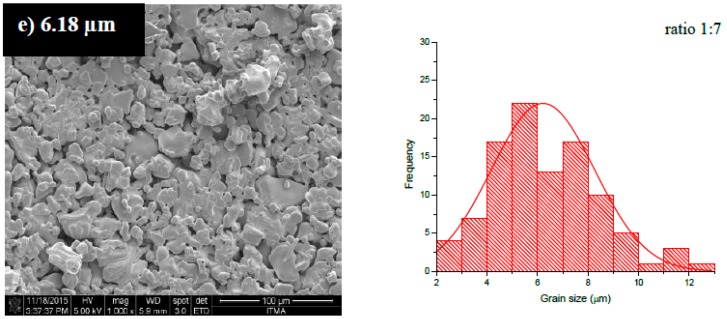
FESEM images of BaFe_12_O_19_ sintered at 1300 °C with ratios of (**a**) 1:3, (**b**) 1:4, (**c**) 1:5, (**d**) 1:6, and (**e**) 1:7.

The FESEM micrographs and the average grain size distributions are shown in Figure 4a–e. As confirmed by the grain size distribution histograms, a large distribution of grain sizes was produced, and all of the distributions were generally in the range of 2–12 µm [13]. Based on the FESEM image, for all samples, some of the grains had nearly spherical shapes, and others had elongated shapes [56,57,58,59]. The grains of the samples appeared to stick each other and agglomerate at different masses throughout the image. The sample with the least powder-to-salt weight ratio had a small grain size compared to the other sample. This was because, at higher salt contents, an increase in grain size due to an increase in the mean particle separation during the salt melt synthesis was produced, leading to enlarged growth without the hindrance of the concomitant growth of surrounding particles or the fusion of particles onto each other [30,37].

Diffusion is a fundamental process that can happen during sample preparation. By applying heat onto the solution, the particles are enabled to be always in contact with each other atomically, and due to microcrystalline grains, required diffusion distances are minimized. During the reaction, the molten salt acted as a nucleation site and controlled the morphology and crystal orientation of the ferrite particles. Also, due to the formation of defects during synthesis, the diffusion may be high. In addition, by the formation of micron-crystalline particles, diffusion can hardly take place through the migration of grain boundaries.

**Figure 5:**
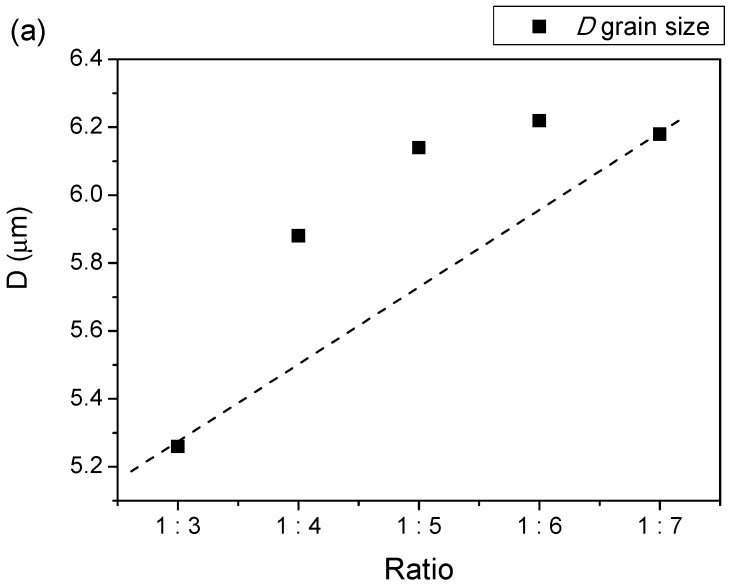

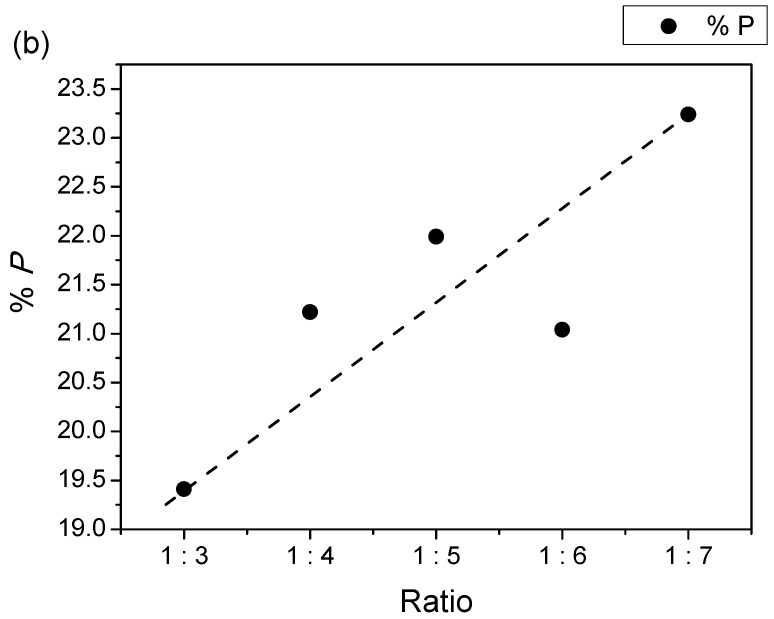
Relationship of (**a**) grain size, *D*, and (**b**) percentage of porosity, %*P* at various ratios of BaFe_12_O_19_.

It can be seen that AN addition enhanced the growth rate of the reactant materials, thus leading to enlargement of the grain size. It was also found that a large amount of AN salts also led to an increase of pore formation (Figure 5a,b).

### 3.3. Magnetic Hysteresis

**Figure 6:**
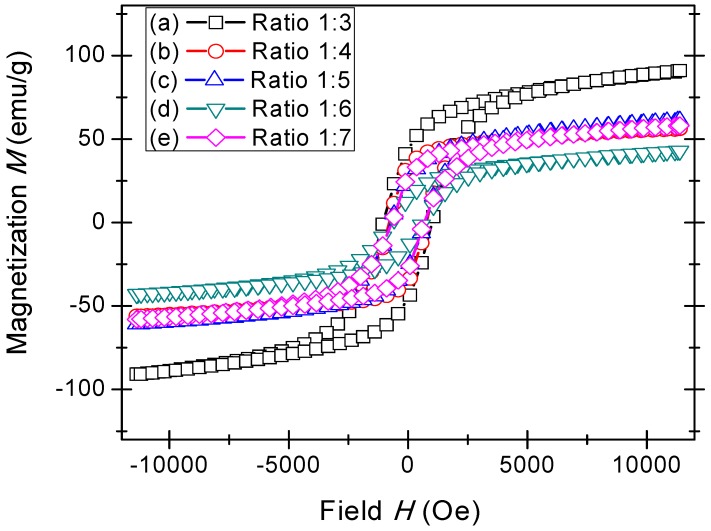
Hysteresis loop of BaFe_12_O_19_ at different ratios (**a**) 1:3, (**b**) 1:4, (**c**) 1:5, (**d**) 1:6, and (**e**) 1:7.

Figure 6 shows the magnetization *M*—magnetic field *H* hysteresis loop of the BaFe_12_O_19_-sintered samples, and the magnetic parameters measured in the field of 10,000 Oe are presented in Table 4. The hysteresis graph shows the effect of the magnetic parameters; coercivity *H*_c_, saturation magnetization *M*_s_, and remanence *M*_r_, with increasing AN contents. It could be observed that the hysteresis loop confirmed that a strong interaction of magnetic moments within domains occurred due to exchange forces where hysteresis loops with a sigmoid shape could be observed. This observed behavior could be considered to be the formation of the ordered magnetism in the sample. In fact, to obtain an ordered magnetism and a well-formed *M*–*H* hysteresis loop, there must exist a significant domain formation, a sufficiently strong anisotropy field, *H*_a_, and optional addition contributions that come from defects such as grain boundaries and pores [9,60].

**Figure 7:**
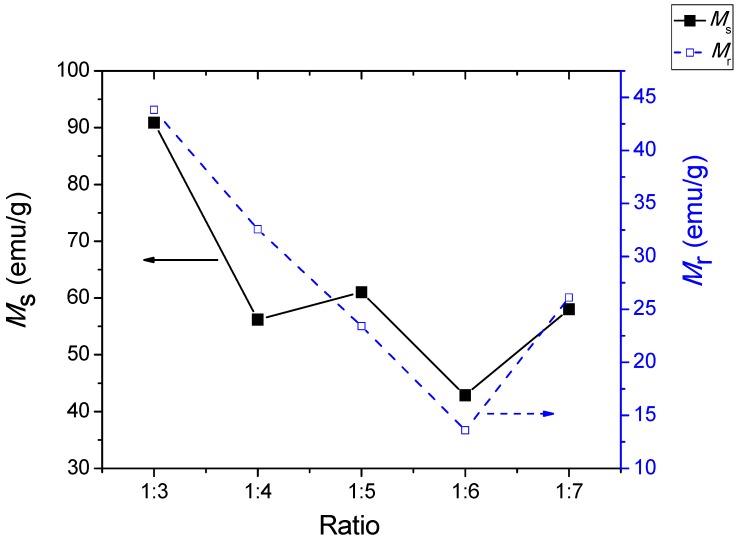
Variation of saturation magnetization, *M*_s_, and remnant, *M*_r_, of BaFe_12_O_19_ at different ratios.

The magnetic parameters *H*_c_, *M*_s_, and *M*_r_ (Figure 6 and Figure 7) decreased with increasing AN contents. These results showed that the addition of salts influenced the magnetic properties of the BaFe_12_O_19_ powders. Large amounts of AN salts yielded a large amount of diamagnetic substances in a ferromagnetic BF system. A sample ratio of 1:3 produced maximum magnetic properties with the values of *H*_c_ being 1317 Oe, *M*_s_ being 90.85 emu/g, and *M*_r_ being 43.83 emu/g, which was higher compared to other methods for synthesizing BaFe_12_O_19_ [4,61,62,63]. The value of magnetization, *M*_s_, of the sample ratio 1:3 was higher than that of single crystals (*M*_s_ is 72 emu/g) due to the formation of high-weight fractions of magnetic phases in the samples. The high values of the magnetic parameters *M*_r_, *M*_s_, and *H*_c_ were associated with structural properties, particularly the phase composition of BaFe_12_O_19_ powders, as well as the microstructure of the samples [4].

**Figure 8:**
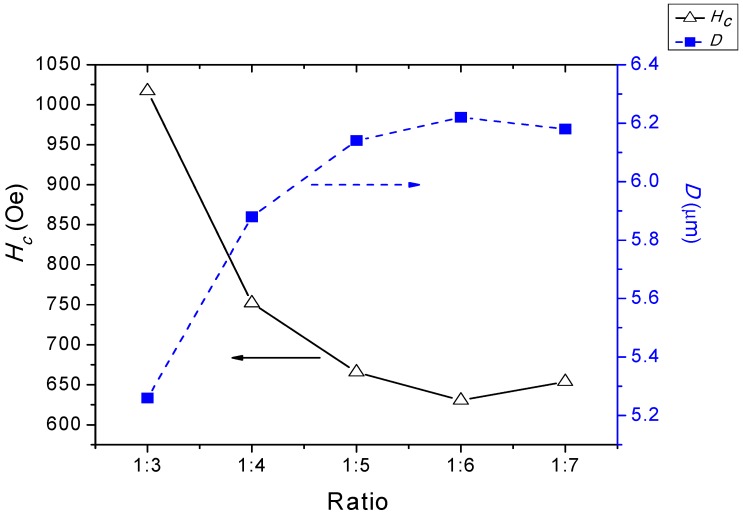
Grain size, *D*, and coercivity, *H*_c_, of BaFe_12_O_19_ at different ratios.

The increased amounts of AN salts resulted in a reduction of the magnetic properties and an enlargement of the grain size [38] (Figure 8). The reduction of the magnetic properties, particularly the *M*_s_ values, were predominantly attributed to the intrinsic factors, which were the degrees of crystallinity and crystalline phases that existed in the samples. It was clearly observed before the Figure 1 that the intermediate Fe_2_O_3_ phase that existed in the samples had ratios of 1:5 and 1:6. However the *M*_s_ value was insignificantly affected, for a sample having a ratio of 1:5, due to only a minute amount of Fe_2_O_3_ being present in the sample, besides having a high intensity of the main crystalline peaks, as compared to that of the sample having a ratio of 1:6. Therefore, a reduction of *M*_s_ could be observed in the sample, having a ratio of 1:6, resulting from the reduction in the crystallinity and the significant existence of the intermediate Fe_2_O_3_ phase.

While *M*_s_ was strongly affected by the intrinsic factors, *H*_c_ also depended on the degree of crystallinity and magnetic anisotropy, but most importantly on the microstructure properties. The latter progress indicated that the grain size strongly affected the resulting magnetic properties [64,65,66], which was also demonstrated by the samples in this study (see Table 4).

Dho et al. [64] stated that the pinning of magnetization at the grain boundaries was the most likely cause for determining the coercivity. Furthermore, with larger grain size, the existence of size-shape anisotropy was negligible, and the coercivity caused by grain boundary pinning would be insignificant, as each grain is in the multi-domain state. It is known that a few micron sizes are large enough to be a multi-domain state, and the coercivity caused by intrinsic factors within the grains should be very small. This results in the coercivity being more affected by the grain size [20].

**Figure 9:**
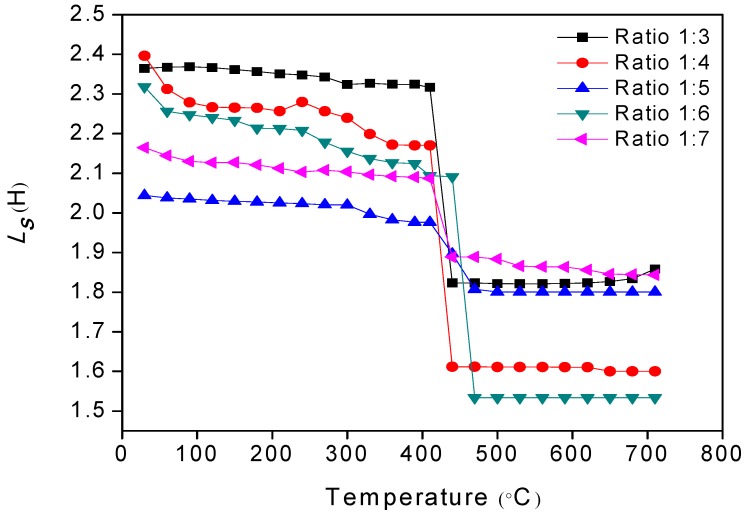
Curie temperatures of samples at different powder-to-salt weight ratios.

Figure 9 shows the graph of inductance, *L_s_*, versus temperature, which determines the Curie temperature, *T_c_*, of all samples. It can be observed that all of the samples experienced a transition from a ferromagnetic to a paramagnetic state above *Tc*. The *Tc* for the powder-to-salt weight ratio of 1:3, 1:4, 1:5, 1:6, and 1:7 were 440, 438, 465, 460, and 444 °C, respectively. These values were very close to 450 °C, the value presented in literature [1,5]. The sample powder-to-salt weight ratios of 1:5 and 1:6 had greater *Tc* values because of a Fe_2_O_3_ phase in the sample, as shown in XRD analysis. This can be explained by the increase in the amount of Fe ions, where there is also an increase in the exchange interaction of the ions [67].

The effect of oxygen stoichiometry through oxygen loss that occurred during sintering temperature at 1300 °C would cause the reduction in Fe^3+^ to Fe^2+^, in order to stabilize the composition after oxygen loss. The strength of the exchange interaction was no longer the same, since more oxygen was missing from the structure. The formation of higher amounts of Fe^2+^ also contributed to the mobility of the electron between Fe^2+^ and Fe^3+^. Consequently, the reduction of Fe^3+^ to Fe^2+^ would affect the interaction, therefore reducing the magnetization, since the net magnetic moment in the barium hexaferrite sample was contributed to by the Fe^3+^ interaction. Stoichiometric changes were also observed through the Curie temperature and the *Tc* measurement, whereas the *Tc* value was highly related to the composition or the stoichiometry inside the sample. It is known that the *Tc* is depends on the exchange interaction between the magnetic ions in the sample [68]. Thus, the presence of the α-Fe_2_O_3_ and γ-Fe_2_O_3_ phases in the samples affects the Curie temperature [69]. The discrepancy of the Curie temperature is not affected by the microstructural changes, because it is strongly affected by the crystal structure and the compositional stoichiometry [70].

### 3.4. AC Conductivity

**Figure 10:**
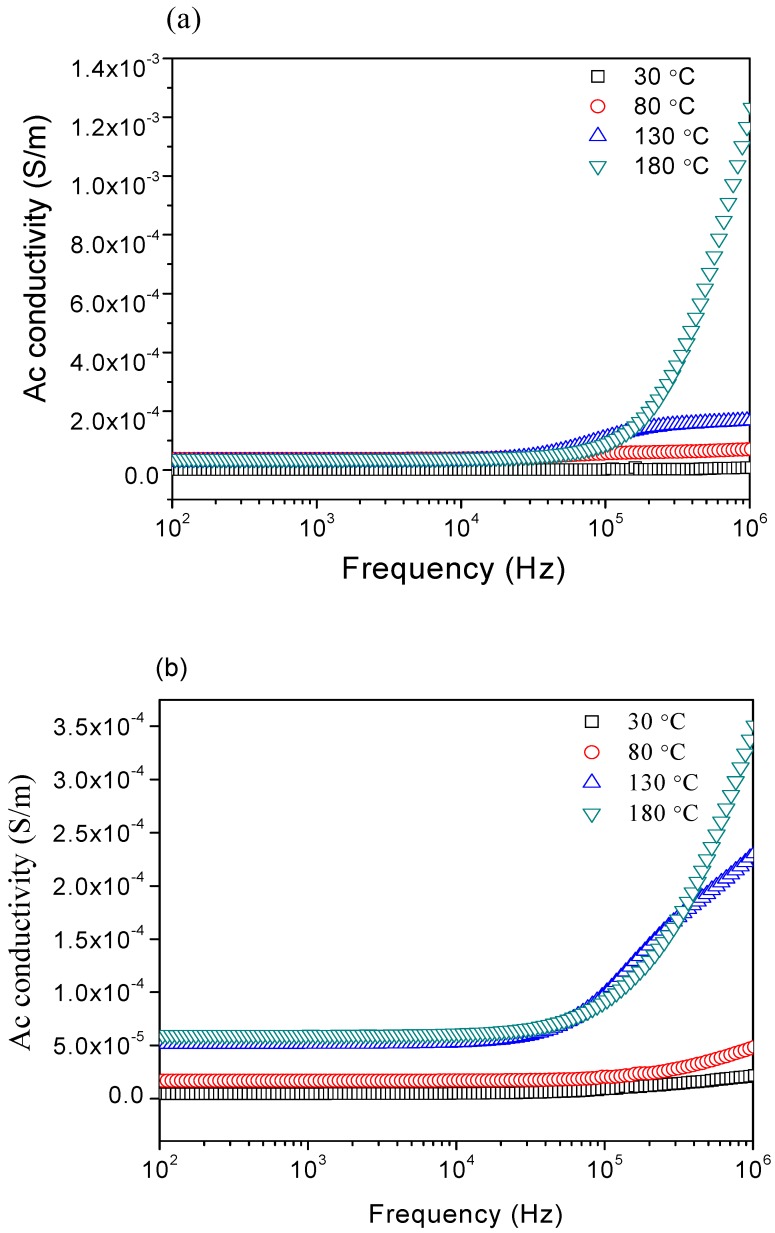

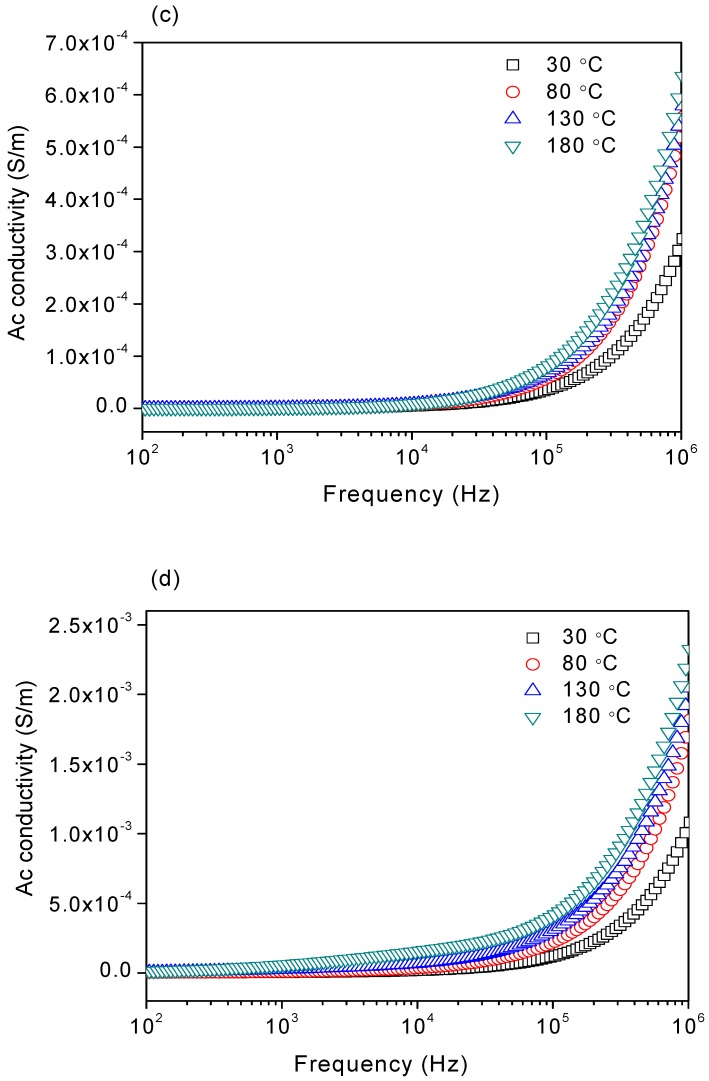

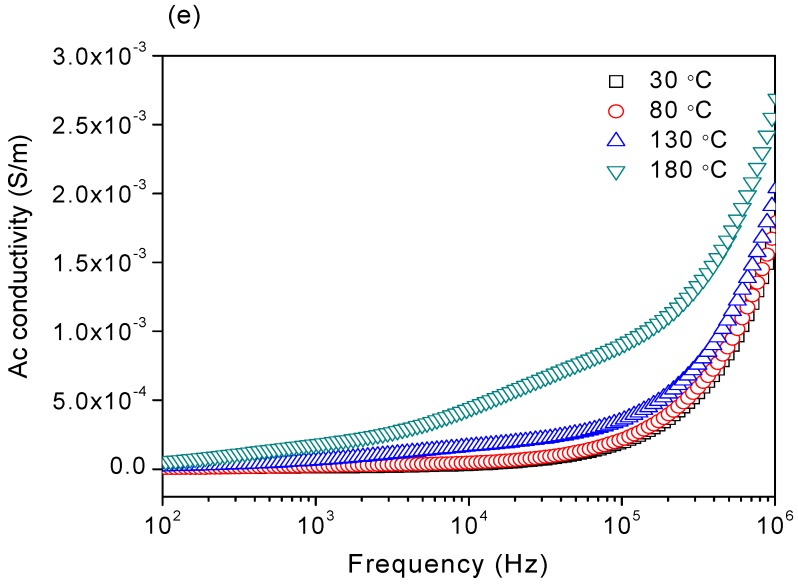
Variations of AC conductivity with respect to the frequency of BaFe_12_O_19_ at different ratios (**a**) 1:3, (**b**) 1:4 (**c**) 1:5 (**d**) 1:6, and (**e**) 1:7.

The variation of AC conductivity (*σ*_AC_) with frequency *f* in the log scale (*f* = 40–10^6^ Hz) at different temperatures (303–453 K) is shown in Figure 10a–e. The experimental *σ*_ac_ conductivity was well-fitted with Johnscher’s power law. The AC conductivity increased with the frequency as the temperature increased. It was highly temperature-dependent [13,14,15]. According to Johnscher’s universal power law, the frequency independence plateau from 40 Hz to 10 kH was attributed to DC-like conductivity. The data analysis showed that the conductivity increased at a high frequency (10^7^ Hz), where the results showed that the conductivity was not temperature-dependent, but frequency-dependent. The conductivity remained almost constant in the low-frequency region, but exhibited dispersion for higher frequencies (*f* > 10^4^ Hz) [13,14,15]. The effect of diamagnetic substitution in barium hexaferrite will have an effect on the slow decrease at low frequency in the dielectric constant value. The level of diamagnetic substitution also results in a peak near 50 GHz in the real and imaginary parts of permeability [7].

The dispersion in conductivity with frequency has been explained by the Koops theorem [71], where the ferrite is composed of two layers: grain with high conductivity, and a grain boundary with poor conductivity. At lower frequencies, grain boundaries are effective with high resistance, giving a constant conductivity; at higher frequencies, the increase in conductivity with the frequency is due to the grain effect, as well as the increase in the hopping of the charge carriers Fe^2+^ ↔ Fe^3+^ at the adjacent octahedral sites. Due to the improvement in the hopping process of the electron, it causes the conductive grains to become more active, thus increasing the conductivity [72,10]. The hopping depends on the local displacement of charges and the concentration of the ferric and ferrous ions at the octahedral sites.

Generally, the increase in AN salts led to larger grain; thus, resulting in higher conductivity (Table 5). The larger grains led to thinner grain boundaries, with less atoms that were in a state of disorder and defect in the material. With fewer amounts of defects that blocked the carrier transition, it resulted in a decrease in resistivity, and an increase in conductivity. Thus, it is probable that BaFe_12_O_19_ has a higher electrical conductivity, due to the ratio of the volume of BaFe_12_O_19_ and the decrease in the disorder.

## 4. Conclusions

BaFe_12_O_19_ were successfully prepared by ammonium nitrate variation in the salt melt technique derived from steel wastes. XRD analysis studies confirmed that the synthesized BaFe_12_O_19_ formed the hexagonal structure. The FTIR characteristics peaks appeared at 542.71 ${\mathrm{cm}}^{-1}$ and 432.48 ${\mathrm{cm}}^{-1}$ which confirmed the metal–oxygen bending, and the vibration of octahedral sites of BaFe_12_O_19_. Increasing the powder-to-AN salts ratio has been observed to produce larger grain sizes, with less grain boundaries in the samples. The samples with a powder-to-salt weight ratio of 1:3 were found to produce the highest coercivity, saturation magnetization, and remnants, with a value of 1317 Oe, 91 emu/g and 44 emu/g, respectively. Addition of diamagnetic salts, which increase the salt ratio, reduced the magnetic properties of the samples. The presence of small amounts of Fe_2_O_3_ in some samples increased the Curie temperature, *T_c_*, of the sample, due to the increase in the exchange interaction between the Fe ions. AC conductivity is a thermally activated process, since the AC conductivity increases with an increase in frequency as the temperature rises. This research work has highly potential for its low cost, and can be used to recycle approached permanent magnet fabrications used in motors, loudspeakers, and electromagnetic (EM) absorber applications.

## Figures and Tables

**Table 1:** X-ray diffraction parameters of the main peak (114), position, *d*-spacing, and height.

Sample	Peak (*hkl*)	Position 2θ(^o^)	*d*-Spacing	Phase	Reference Code	Space Group
1:3	114	34.304	2.62631	Barium hexaferrites	98-001-9939	*P63/mmc*
1:4	114	34.137	2.62631	Barium hexaferrites	98-001-9939	*P63/mmc*
1:5	114	34.160	2.62631	Barium hexaferrites	98-001-9939	*P63/mmc*
	104	33.331	2.68455	Hematite	98-004-6404	*R-3 c*
1:6	114	34.335	2.62631	Barium hexaferrites	98-001-9939	*P63/mmc*
	104	33.337	2.68455	Hematite	98-004-6404	*R-3 c*
1:7	114	34.130	2.62631	Barium hexaferrites	98-001-9939	*P63/mmc*

**Table 2:** Lattice constants *a* and *c*, *c*/*a* ratio, volume of unit cell, *V*, particle size, *D*, X-ray density, *ρ*_x_, bulk density, *ρ*_b_ and porosity, %*P* for BaFe_12_O_19_.

Samples	Lattice Parameter		*c*/*a*	*V* (Å)^3^	*ρ*_x_ (g/cm^3^)	*ρ*_b_ (g/cm^3^)	%*P*
	*a* (Å)	*c* (Å)					
1:3	5.8563	23.0784	3.9408	685.4412	5.3847	4.3393	19.41
1:4	5.8883	23.2048	3.9408	696.7477	5.2974	4.1730	21.22
1:5	5.8829	23.1819	3.9406	694.7841	5.3123	4.1440	21.99
1:6	5.8509	23.0733	3.9435	684.0265	5.3959	4.2603	21.04
1:7	5.8891	23.2048	3.9403	696.9371	5.2959	4.0650	23.24

**Table 3:** Summary of the major vibration modes of BaFe_12_O_19_ at different ratios after sintering.

Samples	Wave Number (cm^−1^)	
1:3	432.48	542.71
1:4	420.14	548.82
1:5	419.62	563.90
1:6	419.13	569.63
1:7	417.62	569.93

**Table 4:** Saturation magnetization *M*_s_, coercivity *H*_c_, and remanence *M*_r_, of BaFe_12_O_19_ at different ratios.

Samples	*M*_s_ (emu/g)	*H*_c_ (Oe)	*M*_r_ (emu/g)	*M*_r_/*M*_s_
1:3	90.87	1317.0	43.83	0.49
1:4	56.18	752.02	32.57	0.59
1:5	61.01	665.67	23.42	0.40
1:6	42.89	630.51	13.58	0.32
1:7	58.00	653.85	26.12	0.46

**Table 5:** Direct current (DC)-like conductivity extracted from the AC conductivity spectrum at different ratios for BaFe_12_O_19 at_ 1 kHz.

Sample	*σ*_dc_ (Ω^−1^m^−1^)			
	30 °C	80 °C	130 °C	180 °C
1:3	1.58 × 10^−6^	3.78 × 10^−5^	2.85 × 10^−5^	3.86 × 10^−5^
1:4	4.53 ×10^−6^	1.69 × 10^−5^	5.13 × 10^−5^	6.01 × 10^−5^
1:5	7.39 × 10^−7^	8.29 × 10^−7^	9.85 × 10^−7^	1.31 × 10^−6^
1:6	3.71 × 10^−6^	9.72 × 10^−6^	2.53 × 10^−5^	5.96 × 10^−5^
1:7	1.35 × 10^−5^	2.41 × 10^−5^	6.40 × 10^−5^	1.83 × 10^−4^
